# Outcomes of an App-Based Intervention to Target Naming Among Individuals With Poststroke Aphasia: Virtual Randomized Controlled Trial

**DOI:** 10.2196/67711

**Published:** 2025-08-18

**Authors:** Esther S Kim, Laura Laird, Carlee Wilson, Steven Stewart, Philip Mildner, Sebastian Möller, Raimund Schatz, Robert P Spang, Jan-Niklas Voigt-Antons, Elizabeth Rochon

**Affiliations:** 1 Department of Communication Sciences and Disorders, University of Alberta, 2-70 Corbett Hall, Edmonton, AB, T6G 2G4, Canada, 1 7804925980; 2 Department of Speech-Language Pathology, University of Toronto, Toronto, ON, Canada; 3 KITE Research Institute, Toronto Rehabilitation - University Health Network, University of Toronto, Toronto, ON, Canada; 4 Nuromedia GmbH, Cologne, Germany; 5 Quality and Usability Lab, Technische Universitat Berlin, Berlin, Germany; 6 Center for Technology Experience, AIT Austrian Institute of Technology, Vienna, Austria; 7 Immersive Reality Lab, Hamm-Lippstadt University of Applied Sciences, Hamm, Germany

**Keywords:** aphasia, rehabilitation, speech-language pathology, app-based therapy, user-centered design, mHealth, adaptive software, technology-based intervention, speech, language, communication, stroke, randomized controlled trial, mobile app, efficacy, quality of life

## Abstract

**Background:**

People with aphasia present with language and communication deficits, most notably in lexical retrieval (naming). Although positive outcomes in naming have been observed following speech-language treatment, many individuals with aphasia continue to face impairments after the acute phase of rehabilitation. Mobile app–based therapies are increasingly being used by speech-language pathologists in the rehabilitation of people with aphasia as an adjunct to or in lieu of traditional in-person therapy approaches. These apps can increase the intensity of treatment and have been shown to result in meaningful outcomes across several domains.

**Objective:**

VoiceAdapt is a mobile therapy app addressing naming impairments, designed within a user-centered design framework. The VoiceAdapt app uses two evidence-based lexical retrieval treatments to engage people with aphasia to improve their naming abilities through interaction with the app. The purpose of this study was to conduct a randomized controlled trial to examine the preliminary clinical efficacy of training with VoiceAdapt on the language and communication outcomes of people with aphasia.

**Methods:**

A two-arm, waitlist-controlled, crossover group randomized controlled trial was conducted at two sites within Canada. During the intervention phase, participants completed 5 weeks of independent training with the app, which involved naming practice using Semantic Features Analysis and Phonological Components Analysis. The primary outcome measure was naming performance (Boston Naming Test); secondary outcomes included measures of overall language and naming (Western Aphasia Battery-Revised), communication (Communication Effectiveness Index), and quality of life (Stroke and Aphasia Quality of Life Scale-39).

**Results:**

A total of 37 people with aphasia in the chronic stages (average 4.6 y postonset of aphasia) participated in this study. Participants used the app for an average of 20 hours over the 5-week intervention phase. Training with VoiceAdapt resulted in an increase of 1.6 points on the Boston Naming Test (Cohen *d*=0.3). Evidence for improved naming was also observed on trained items, as well as subtests of naming or word-finding on the WAB-R. Training with the app also resulted in a significant increase in participants’ perceptions of their communication quality of life (increase of 0.1 points; Cohen *d*=0.3), but no other measures (WAB-R Aphasia Quotient, Communicative Effectiveness Index) were significant.

**Conclusions:**

Individuals with aphasia who used the VoiceAdapt app for 5 weeks to target naming skills demonstrated measurable gains in naming and communication-based quality of life. Notably, these changes were observed in a remotely delivered program, in participants who were in the chronic stages of aphasia. These findings inform the profession on the use of app-based home therapy programs as an accessible, cost-effective option for individuals in the chronic stages of recovery who often have limited options for rehabilitation.

## Introduction

### Background

Aphasia, an acquired language disorder most commonly caused by stroke, impacts linguistic expression and comprehension in both written and oral modalities. People with aphasia may have impairments in their ability to speak, understand, read, and write. Thus, aphasia can severely limit activities and restrict participation in everyday activities [1]. Nearly one-third of individuals who experience a stroke have aphasia [2,3], and its presence has been associated with poorer outcomes in both acute and chronic periods following stroke [4,5].

Speech-language pathologists (SLPs) provide rehabilitation services for people with aphasia, often focusing on remediation of the language impairment or training of compensatory strategies. Such interventions have been shown to improve outcomes for people with aphasia [6,7], but constraints on health care resources often prevent people with aphasia from receiving these services, especially in the chronic stages. Recently, the proliferation of mobile app–based therapies available to deliver speech-language interventions has meant that people with aphasia can receive prolonged rehabilitation in an efficient, cost-effective manner. SLPs have increasingly been using such apps as adjuvant therapy approaches, or even in lieu of traditional (face-to-face) therapy, with positive outcomes observed across several domains [8,9]. One benefit of these apps is that people with aphasia can increase the intensity of their rehabilitation, a factor that is associated with greater long-term recovery [7,10-12]. In addition, app-based interventions can be self-delivered by people with aphasia and still yield positive language and communication outcomes [13-15]. Moreover, app-based interventions allow for therapy content to be adapted according to the user or patient state, due to the ability to measure multiple parameters of user interactions with touch-based devices.

The research evidence investigating the feasibility, acceptability, and clinical utility of tablet-based speech-language therapy apps for aphasia has increased in recent years, with inconclusive results. In part, this may relate to the chronicity of individuals with aphasia included in these studies. For instance, Ruiz et al [16] found no difference between an intervention group provided with tablet-based therapy in the first week poststroke and a no-treatment control group. However, Mallet et al [17,18] reported that the use of mobile therapy apps for speech-language therapy was feasible in the very early stages of acute stroke recovery and could potentially be used to bridge the gap between discharge from acute care and the start of outpatient therapy. On the other hand, evidence is emerging on the utility of app-based therapy for adults in the chronic stages of acquired aphasia. Across a growing number of studies, individuals with aphasia have shown improvement across a variety of domains, including word production, reading, naming, spoken discourse, quality of life, and overall language [13-15,19-24].

App-based therapy has the potential to increase treatment dosage (total hours), intensity (hours per week), frequency (days per week), and duration (total weeks)—important factors that contribute to positive speech-language therapy outcomes [25]. For instance, participants in a study by Alam et al [22] were found to benefit more from therapy in the condition where an app was provided as an adjunct to therapy (the “study” arm) than in the condition where there was no adjunct (the “control” arm). However, it should be noted that participants in the study arm received more therapy overall due to the addition of the app. Individuals with aphasia who self-managed their own participation using therapy apps in the absence of face-to-face therapy have also demonstrated improved language outcomes [13-15,21]. Indeed, app-based therapy in lieu of traditional therapy, particularly for individuals in the chronic stages of aphasia, could be an important component of optimizing recovery in the context of limited rehabilitation resources. However, further research to understand the impact of apps (alone and in combination with face-to-face rehabilitation) is warranted.

A recent review by Szeto et al [26] on the effect of mobile apps for stroke rehabilitation reported that aphasia therapy apps that resulted in the greatest improvements were ones that incorporated characteristics of face-to-face therapy. These included massed practice (repetition) of goal-oriented, task-specific exercises, user interactivity, and multisensory stimulation. A similar review identifying and evaluating mobile apps for speech therapy in adults with communication disorders (not limited to aphasia) revealed that there was a general lack of engaging and interactive elements in the apps [27]. Increasing the usability of therapy apps could be enhanced by designing apps with input from relevant stakeholders, following the principles of user-centered design (UCD). UCD is a systematic approach to meeting user experience goals and usability through integrating the needs and abilities of end users during the design process [28]. Although the number of therapy apps designed for aphasia is steadily increasing [27,29], very few have been designed following the principles of UCD [30,31], despite increasing evidence supporting the beneficial impacts of UCD on user experience in digital health technologies [32]. Indeed, in their review of 70 therapy apps for adults with communication disorders, Vaezipour et al [27] recommend using a co-design process involving end users (ie, people with aphasia), subject matter experts (ie, SLPs), and incorporating interactive elements and gamification to tailor the app to individual needs.

### Development of the VoiceAdapt App

The VoiceAdapt app, developed by an international team of interdisciplinary researchers from the fields of computer science, usability design, and speech-language pathology, is a tablet-based app focused on improving naming abilities in individuals with aphasia. VoiceAdapt instantiates two evidence-based lexical retrieval protocols, Phonological Components Analysis (PCA) [33,34] and Semantic Feature Analysis (SFA) [35,36], into an adaptive, voice-responsive therapy app that people with aphasia can use to improve their naming skills.

Using UCD principles, the team engaged 75 end users (including people with aphasia, clinicians, and caregivers) in designing the app. Over the course of three iteration cycles, end users participated in interviews to elicit user requirements, provided feedback on mock-ups and prototypes, and evaluated the designs with a focus on user experience of multimodal speech and adaptivity [32]. The end result was a mobile speech-language training app for iOS and Android that supported regular training sessions, adaptivity, personalization, and addressed multimodal language goals (reading, understanding, speech production).

### Objective

The purpose of this study is to report the efficacy of a beta version of the VoiceAdapt app, within a randomized controlled trial (RCT; ClinicalTrials.gov NCT04108364). Specifically, we examined the impact of training with the VoiceAdapt app on language and communication outcomes in individuals with chronic aphasia. Primary outcomes were naming ability, and secondary outcomes included measures of overall language, communication effectiveness, and quality of life.

## Methods

### Recruitment

Participants were recruited offline between August 2020 and August 2022 in two geographic regions in Canada through the Language Sciences Lab (Department of Speech-Language Pathology, University of Toronto) and Aphasia Research Lab (Department of Communication Sciences and Disorders, University of Alberta), based in Toronto, Ontario, and Edmonton, Alberta, respectively. Information about the study was disseminated to clinicians and programs serving individuals with aphasia. Interested participants reached out directly to study personnel (CW, Edmonton; LL, Toronto) and provided informed consent prior to being enrolled in the study. Communicatively accessible consent forms were used, and all study personnel were trained in supported communication techniques.

Participants enrolled in the study were at least 6 months postonset of a stroke in the left hemisphere, had aphasia of mild to moderate severity (Aphasia Quotient ≥30 on Western Aphasia Battery-Revised [WAB-R] [37]), scored below 75% correct on the Boston-Naming Test [38] at initial assessment, had a prominent verbal expression impairment (based on assessment scores and SLP determination), passed screenings of basic sensory (vision and hearing) and cognitive functioning, and spoke English as a primary language. Participants with severe aphasia were excluded from the study due to significant verbal expression impairment and limited naming abilities in this population. As the app was designed for self-directed treatment, individuals with severe aphasia profiles were unlikely to benefit from this type of intervention. Participants also had access to a computer or tablet and the internet in order to participate in web-based assessment sessions. In addition to meeting all inclusion criteria, participants were excluded from participation if they were currently engaged in individual (one-on-one) speech-language therapy or were currently using speech-language therapy apps. All study procedures (intake, informed consent, and assessments) took place using videoconferencing software, in line with recent advancements indicating the feasibility and reliability of virtual assessment protocols [39,40].

Planned enrollment for the study was 80 participants based on sample size calculations, with interim analyses planned when quarterly enrollment targets were met (ie, 20, 40, and 60 participants) [39]. The sample size calculation was based on medium effect sizes estimated in this study according to the results shown by Varley et al [41]. Group sample sizes of 72 are needed to achieve 80% power to detect a difference in proportions of 0.25 between the null hypothesis and the alternative hypothesis using a 1-tailed *t* test with a probable α error of 0.05. Accounting for a dropout rate of approximately 10% (based on the dropout rate of Varley et al [41]) from baseline to follow-up, we estimated we would need 79 participants (rounded up to 80). It was determined that the trial would be stopped early if evidence of a treatment effect on the primary outcome measure was observed at the time of interim analyses.

### Design

**Figure 1.**
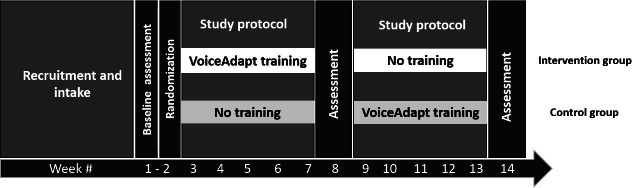
Study design.

The RCT was a two-arm, randomized, waitlist-controlled, crossover group design. All participants completed initial assessments and then were randomized into intervention or treatment-deferred control groups. Participants in the intervention group completed training with the VoiceAdapt for 5 weeks during Phase 1 of the study, while those in the control group continued with usual care. Following midpoint assessments for all participants, those in the treatment-deferred control group crossed over into receiving treatment, while those in the intervention group crossed over into the control phase. Final assessments took place following Phase 3, approximately 13 weeks following study enrollment (Figure 1). Although the study was originally intended to be conducted in person, the COVID-19 pandemic necessitated a move to all procedures being conducted virtually.

An individual not affiliated with the study created a computer-generated randomization sequence using Excel (version 2007; Microsoft Corp), stratified by site with 1:1 allocation using random block sizes of 10. Each cell containing the allocation status was concealed using black fill in the spreadsheet. One member of the study team (CW) revealed allocation status to participants (Edmonton site) or to the research coordinator at the Toronto site (LL), who then related the status to participants following baseline assessment. Assessments were completed by SLPs (Edmonton site) or speech-language pathology graduate students under the supervision of a SLP (Toronto site) who were blinded to the treatment condition of participants. Participants were told not to reveal to assessors which group they were in.

### Intervention

The VoiceAdapt app intervention comprises naming exercises based on SFA and PCA, two evidence-based anomia treatments, delivered on a mobile tablet. A color picture is presented along with a series of prompts designed to engage the people with aphasia in providing semantic features (eg, “What is it used for?” [Action]; “What does it look like?” [Properties]), or phonological components (eg, “What sound does it start with?” [First sound]; “What is a rhyming word?” [Rhyme]). People with aphasia randomized to the VoiceAdapt app treatment group were instructed to practice using the app for 1 hour per day every weekday (5 days per week) for 5 weeks for a total of 25 hours of intervention. Participants were provided with tablets with the app loaded, or could download the app from a link provided by the researchers onto their own devices. Participants selected interests at initial app setup, which then tailored the practice items to each individual. Research staff provided orientation to the app and monitored participants’ app use (number of minutes per week) through the app portal. Research staff (CW and LL) checked in with participants through email, or phone, or video calls on a weekly basis, providing practice reminders and answering questions.

### Outcome Measures

The primary outcome measure was the Boston Naming Test (BNT) [38] to measure naming, secondary outcome measures were WAB-R [37] and Stroke and Aphasia Quality of Life Scale (SAQOL-39g) [42] to measure quality of life, and Communication Effectiveness Index (CETI) [43] to measure the person with aphasia’s perceptions of communication effectiveness. Both the person with aphasia and their caregiver (where applicable) completed the CETI. These outcomes were assessed at baseline and after intervention phases (Figure 1). In addition, at the final assessment session, the Study Stimulus Naming Test (SSNT), a measure of performance on trained and untrained items on the VoiceAdapt app, the System Usability Scale (SUS) [44], and a 5-question posttreatment questionnaire specific to app use were administered to all participants.

### Fidelity and Reliability

To ensure fidelity of procedures across both sites, a standardized intake and assessment training protocol was developed. Assessors at both sites completed all steps in the training protocol including reading background papers [39,40], reviewing intake and consent forms and manuals for all assessments, observing the study coordinator or primary investigators (licensed SLPs) doing a complete assessment session, and finally, being observed by the study coordinators or primary investigators to ensure protocol adherence prior to completing assessments on their own.

Administration of the primary outcome measure (BNT) was video recorded through the Zoom (Zoom Video Communications, Inc) videoconferencing platform. A total of 16 assessment sessions (just under 15% of the sample) were randomly selected for assessing the reliability of scoring. Point-to-point reliability was high overall (Cohen κ=0.91).

### Data Management

Study data were collected and managed using REDCap (Research Electronic Data Capture; Vanderbilt University) electronic data capture tools hosted at the University of Alberta [45,46]. REDCap is a secure, web-based software platform designed to support data capture for research studies, providing (1) an intuitive interface for validated data capture, (2) audit trails for tracking data manipulation and export procedures, (3) automated export procedures for seamless data downloads to common statistical packages, and (4) procedures for data integration and interoperability with external sources.

### Ethical Considerations

This project was approved by the University of Toronto Health Sciences Research Ethics Board (Protocol Number 37980) and the University of Alberta Health Research Ethics Board (Pro00091587). All participants provided informed consent following an explanation of research procedures in a communicatively accessible manner by research staff who were trained in supported communication techniques. All data were anonymized and deidentified. Participants were not provided with monetary compensation for their participation in this study.

## Results

### Participants

**Figure 2.**
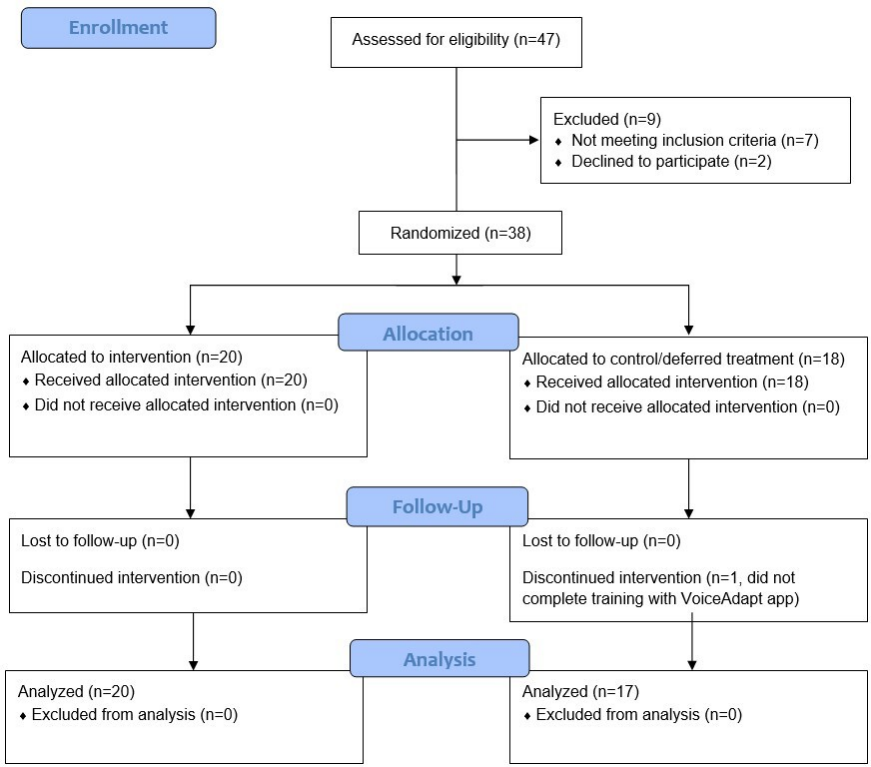
Participant flowchart.

A total of 38 participants with aphasia (13 female) were recruited and randomized across the two sites. In total, 20 individuals were randomly assigned to receive training with VoiceAdapt first, while 18 individuals were randomly assigned to the deferred treatment (control) condition. One individual randomized to the control group dropped out of the study shortly after starting treatment due to frustrations with the automatic speech recognition (ASR) aspects of the app. As the withdrawn participant only contributed two baseline assessments and did not contribute any treatment information, their data were not included in analyses. A total of 37 participants completed both phases of the study (Figure 2). The trial ended early due to results from interim analyses, as well as slower-than-expected recruitment due to challenges following the COVID-19 pandemic (see details below). Participants had a mean age of 62.2 (SD 13.9) years and 15.1 (SD 3.2) years of education and were an average of 4.6 (SD 4.2) years postonset of aphasia. No differences in age, education, years postonset, or baseline WAB-AQ/BNT were observed between groups (Table 1). Site differences were present for age and education across sites, with participants recruited through the University of Alberta being younger and having less education than those recruited through the University of Toronto. The number of participants across allocation conditions and mean demographic information are presented in Table 1.

**Table 1.** Participant mean age, education, years post-onset, WAB-AQ^a^, and BNT^b^ scores across allocations^c^.

Characteristic	Intervention (n=20; 6F), mean (SD)	Control (n=17; 7F), mean (SD)	Total (n=37; 13F), mean (SD)
Age (years)	59.9 (15.3)	64.8 (11.9)	62.2 (13.9)
Education	14.7 (2.6)	15.5 (3.7)	15.1 (3.2)
Years postonset	3.5 (2.5)	5.8 (5.3)	4.6 (4.2)
WAB-AQ^a^	68.3 (15.2)	73.6 (14.0)	70.7 (14.7)
BNT^b^	25.0 (12.1)	23.5 (12.5)	24.3 (12.1)

a WAB-AQ: Western Aphasia Battery*-*Revised Aphasia Quotient.

b BNT: Boston Naming Test.

c Intervention refers to participants randomized to receive VoiceAdapt training first; control refers to participants randomized to the treatment-deferred control group.

### Analytic Strategy

The 2×2 crossover trial with one baseline was analyzed using Bayesian inference to estimate the parameters of a mixed-effects regression model. Using mixed-effects regression can account for hierarchical sampling of participants within sites. Using this crossover design allowed estimation of treatment effect, time effect, sequence effect, and carryover effect. Bayesian inference was chosen for accurate multiple comparisons [47]. For each outcome, we modeled treatment effect, time effect, and the outcome at baseline covariate as a normal distribution with unknown variance using a noninformative flat prior for the mean parameter and a noninformative inverse-gamma prior for the variance parameter that was sampled independently of the other parameters. This Bayesian model was fit using the adaptive Metropolis-Hastings Markov chain Monte Carlo simulation method implemented in Stata (version 16; StataCorp LLC). The proportion of the posterior distribution of the treatment effect that was greater than zero was interpreted as the probability that the outcome increased. For such interval hypothesis testing, a probability of 0.95 or higher was considered strong evidence of improvement during VoiceAdapt training. In these data, we found no statistical evidence for a sequence effect or a carryover effect, so these were dropped from our model. We also checked for an effect of app use and found no statistical evidence for an effect, so it was also dropped from the model. All analyses were carried out on the 37 participants who completed both phases of the study unless otherwise indicated.

Our initial sample size calculation was based on using frequentist approaches. There were several realities that necessitated a change to Bayesian analyses. Due to recruitment challenges in the virtual context, we needed to do interim analyses more frequently than outlined in our protocol. Bayesian analysis allows researchers to conduct interim analyses without being penalized because decisions at analysis points are based on the posterior probabilities of treatment effects [47]. Bayesian analysis also provides a straightforward means of analyzing multiple outcomes multiple times. While our power analysis was based on the effect size of one strong primary outcome, BNT, in reality, our different outcomes had differential effects based on our interim analyses. Given that we found significant results with our reduced sample size, and the recruitment challenges referenced above, we decided to end the trial early.

### Primary Outcome

The primary outcome was the BNT to measure change in naming performance as a result of training with the VoiceAdapt app. Participants increased an average of 1.6 points on the BNT (equal-tailed 95% credible interval −0.3 to 3.6; Cohen *d*=0.3) when training with VoiceAdapt (Table 2).

**Table 2.** Median and equal-tailed 95% credible interval of the posterior probability distribution of each parameter of the cross-over model for the primary outcome (BNT^a^).

Parameter	BNT, median (95% credible interval)
Fixed-effect parameters^b^	
VoiceAdapt effect	1.6 (−0.3 to 3.6)^c^
Time effect	1.5 (−0.4 to 3.3)
Outcome at baseline covariate	1.0 (0.8 to 1.1)
Constant	2 (−3 to 6)
Random-effect parameters	
Among participant variance	21 (8 to 39)
Residual variance	17 (10 to 28)

a BNT: Boston Naming Test.

b Proportion of Bayesian posterior probability distribution of VoiceAdapt effect greater than zero, Cohen *d: *0.954*.*

c Cohen *d*=0.3.

### Other Outcomes: Naming Ability

Additional analyses with other measures related specifically to naming abilities were examined further using the same analysis procedures. These included the Object Naming subtest on the WAB-R (20 item naming test); the Naming and Word Finding subscore of the WAB-R (comprising combined scores from Object Naming, Word Fluency, Sentence Completion, and Responsive Speech subsets); and the SSNT, a 322 item naming measure comprising both trained and untrained words programmed into the VoiceAdapt app. As seen in Table 3, training with VoiceAdapt resulted in an increase of 3 points on the Object Naming subtest (equal-tailed 95% credible interval 1-5) and an increase of 2.6 points on the Naming and Word Finding subtest (equal-tailed 95% credible interval 0.15-5), representing small effects (Cohen *d*=0.4 and 0.3, respectively). Interestingly, the SSNT demonstrated the greatest change as a result of training with VoiceAdapt, with a median change score of 13 (equal-tailed 95% credible interval 7-19; Cohen *d*=0.9).

**Table 3.** Median and equal-tailed 95% credible interval of the posterior probability distribution of each parameter of the cross-over model for additional outcomes (SSNT^a^, WAB-R^b^ Object Naming, WAB-R Naming, and Word Finding total).

Parameter	SSNT	Object Naming (WAB-R)	Naming and Word Finding (WAB-R)^b^
Fixed-effect parameters			
VoiceAdapt effect, median (95% credible interval)	13 (7 to 19)^c^	3 (1 to 5)^d^	2.6 (0.15 to 5)^e^
Proportion of Bayesian posterior probability distribution of VoiceAdapt effect greater than zero, Cohen *d*	0.999	0.998	0.980
Time effect, median (95% credible interval)	13 (6 to 19)	−0.6 (−2.6 to 1.4)	−0.6 (−3.0 to 1.9)
Outcome at baseline covariate, median (95% credible interval)	1.05 (0.9 to 1.2)	1.0 (0.8 to 1.1)	1.0 (0.9 to 1.1)
Constant, median (95% credible interval)	1 (20 to 18)	1 (−5 to 7)	1 (9 to 6)
Random-effect parameters, median (95% credible interval)			
Among participant variance	375 (188 to 668)	23 (10 to 43)	31 (11 to 60)
Residual variance	168 (103 to 277)	19 (11 to 30)	28 (17 to 45)

a SSNT: Study Stimulus Naming Test.

b WAB-R: Western Aphasia Battery-Revised.

c Cohen *d*=0.9.

d Cohen *d*=0.4.

e Cohen *d*=0.3.

### Secondary Outcomes

There was no significant effect of VoiceAdapt training on overall language abilities, as evidenced by a mean change in WAB-R Aphasia Quotient of 0.4 (Cohen *d*=0.01). VoiceAdapt training resulted in a significant increase in participants’ perceptions on the SAQOL-39g Communication Quality of Life subscale (increase of 0.1 points; Cohen *d*=0.3), representing a small effect, but not on overall Quality of Life (SAQOL-39g mean score decrease of 0.01 points; Cohen *d*=0.1). Measures of communication effectiveness as measured by the participant (CETI-participant mean score=−5; Cohen *d*=0.1) or their partners (CETI-partner mean score=0.1; Cohen *d*=0.2) were not found to be significantly improved by VoiceAdapt training (Table 4).

**Table 4.** Median and equal-tailed 95% credible interval of the posterior probability distribution of each parameter of the cross-over model for the secondary outcomes (WAB-R^a^ aphasia quotient, SAQOL-39g^b^ mean score*,* SAQOL-39g Communication score, CETI^c^, and CETI-Partner)^d^.

Parameter	WAB-R AQ	SAQOL-39g mean	SAQOL-39Comm	CETI	CETI-Partner
Fixed-effect parameters					
VoiceAdapt effect, median (95% credible interval)	0.4 (−1.1 to 1.8)^e^	−0.005 (−0.1 to 0.1)^f^	0.1 (−0.1 to 0.3)^g^	−5 (−12 to 4)^f^	0.1 (−7 to 7)^h^
Proportion of Bayesian posterior probability distribution of VoiceAdapt effect greater than zero, Cohen *d*	0.707	0.468	0.846	0.142	0.506
Time effect, median (95% credible interval)	0.4 (−1.1 to 1.8)	0.01 (−0.1 to 0.1)	−0.05 (−0.3 to 0.1)	1 (−7 to 10)	9 (2 to 16)
Outcome at baseline covariate, median (95% credible interval)	1.0 (0.9 to 1.1)	0.9 (0.7 to 1.1)	0.8 (0.5 to 1.0)	0.6 (0.4 to 0.9)	0.8 (0.5 to 1)
Constant, median (95% credible interval)	−1 (−7 to 4)	0.4 (−0.4 to 1.2)	0.9 (0.1 to 1.6)	44 (22 to 66)	18 (−3 to 38)
Random-effect parameters, median (95% credible interval)					
Among participant variance	7 (1 to 15)	0.1 (0.05 to 0.2)	0.2 (0.1 to 0.3)	231 (37 to 493)	137 (12 to 311)
Residual variance	10 (6 to 17)	0.06 (0.03 to 0.1)	0.2 (0.1 to 0.3)	309 (175 to 526)	151 (76 to 289)

a WAB-R: Western Aphasia Battery-Revised.

b SAQOL-39g: Stroke and Aphasia Quality of Life Scale.

c CETI: Communication Effectiveness Index.

d Sample includes three longitudinal assessments of 37 participants, except for CETI Partner outcome because 7 participants did not have a participating partner.

e Cohen *d*=0.01.

f Cohen *d*=0.1.

g Cohen *d*=0.3.

h Cohen *d*=0.2.

### Participant Perceptions of VoiceAdapt App

Participants used the app for an average of 19.5 (SD 8.6) hours over the course of the 5-week VoiceAdapt intervention phase. The SUS [44], a 10-item questionnaire, was used to evaluate participant perceptions of the ease of using the VoiceAdapt app. Each question has one of five response options, ranging from strongly disagree to strongly agree; items are scored (accounting for positive or negative valence) and multiplied by 2.5 to yield an overall usability score out of 100. The average SUS score for all participants was 61.2 (SD 18.9). The mean score for each question ranged from 2.2 to 2.6, indicating participants felt neutral or agreed with statements pertaining to the usability of the app. In addition to the SUS, a 5-question posttreatment questionnaire was administered to all participants, asking about their experiences with the app. Mean responses to the posttreatment questionnaire are provided in Table 5.

**Table 5.** Average responses to posttreatment questionnaires by study participants.

Question	Average response (1=strongly disagree; 5=strongly agree)
I really liked the app	4.1
The app was easy to use	3.9
Training with the app helped my communication	3.9
I would use this app if it were still available	3.8
I would be willing to pay a fee for the VoiceAdapt app	3.2

## Discussion

### Principal Findings

This RCT was designed to assess the efficacy of the VoiceAdapt app, a tablet-based naming therapy program for people with aphasia. The app implemented evidence-based treatment principles into an adaptive, personalizable app that addressed multimodal language stimulation with the aim of stimulating language production, specifically single-word naming.

Training with the VoiceAdapt app resulted in improved single-word naming performance. The change in naming after treatment on the primary outcome measure (ie, BNT) was minimal, though statistically robust. Converging evidence for improved naming also came from observed improvements on subtests of the WAB-R related specifically to naming abilities (Naming and Word Finding, Object Naming), as well as the SSNT, a set of 322 items (including trained and untrained words). Notably, improvements on the SSNT were accompanied by a large effect size. These results extend the literature base on the efficacy of SFA and PCA for improving naming [48,49], as well as the growing number of studies reporting positive outcomes from app-based instantiations of these therapy protocols [15,18,23].

Importantly, participants’ perception of their quality of life as it related specifically to their communication abilities (SAQOL-39g Communication score) also revealed a small but significant improvement after VoiceAdapt training, indicating that over and above changes in actual numbers of words produced after training, participants felt more positive about their communicative abilities (eg, speaking in general, finding the right word, and being understood by others). In contrast to the participants’ perceived communication quality of life, communicative effectiveness as measured by the CETI was not perceived to have improved after VoiceAdapt treatment by either the participants or their family members. The concept of communicative effectiveness extends beyond performance on linguistic tasks to situational interactions in the real world [50]. Implementing exercises or activities in future apps that provide the opportunity to improve participation (eg, in conversations) and social integration, perhaps by incorporating digital networking for peer support [51] or virtual reality [52], may in the future lead to generalization to domains of communication effectiveness, overall functioning, and well-being.

Despite improvements in naming and communication quality of life, secondary outcomes encompassing overall language and communication or quality of life did not change significantly. In part, this may be related to the broad domains they were measuring. For instance, the WAB-R AQ calculation includes language comprehension, repetition, and spoken discourse in addition to naming. Similarly, the mean SAQOL-39g score includes evaluation of activities of daily living (eg, preparing food and getting dressed), physical activities (eg, walking and standing), and emotional or mental health items, in addition to communication items. Therefore, despite current recommendations for reporting outcomes for aphasia treatment research [53], it may have been unrealistic to expect change on these two comprehensive measures when only one domain, naming, was implemented in the training app. Incorporating different and varied exercises in the future that target additional language domains may lead to generalization to other language domains.

Recent reviews of the effectiveness of speech-language therapy have reported that intensity (dosage, frequency, and duration) of treatment is associated with the largest gains [25], particularly for individuals in the chronic phases of aphasia. However, in this study, app use was not found to be associated with improvement. In addition, although participants were tasked with practicing for 1 hour daily, average app use was less than that. Nonetheless, our participants, who were an average of 4.6 years (SD 4.2) postonset of aphasia, self-managed their therapy with the app with periodic check-ins from the intervention team, supporting prior reports [15,21,26] that tablet-based therapy apps can be an effective option for people with aphasia in the chronic stages to continue to work on their language [54].

One distinctive feature of the VoiceAdapt app was in its design, incorporating UCD principles with input from end users (therapists, people with aphasia, etc), consistent with findings demonstrating that UCD can significantly enhance user engagement and intervention outcomes in technology-based health apps [27,55]. The app included several features recommended in Vaezipour et al’s [27] review of mobile apps. Specifically, the app was tailored to participants’ interests in that they were able to select categories of items they wished to practice. Participants also interacted with the app to select multimodal cues as needed, providing individualized support according to their performance. Despite this, participants’ ratings of app favorability were in the midrange (mean SUS score 61.2, SD 18.9). Other studies investigating therapy apps have reported higher scores [56,57], but there may be other factors contributing to these higher scores (eg, more clinician involvement and different app content). Nonetheless, improvements could be made to further enhance the effectiveness and usability of the app. One limitation of the app was that the ASR software built into the app was not always accurate, particularly given the common co-occurrence of accompanying motor speech impairments with poststroke aphasia. Indeed, frustrations with ASR contributed to the one participant’s withdrawal we had in this study. As this is a rapidly evolving area of study and development, future iterations of the app may perform better in this regard. Incorporation of additional features demonstrated to improve engagement and interactivity [26,27], such as social interaction and gamification, could also result in an improved app in the future. Importantly, future additions and designs should incorporate the wishes, needs, and recommendations of people with aphasia [31].

### Limitations and Future Directions

The planned methods, including recruitment activities, assessments, and treatment, were all modified to be delivered virtually due to the COVID-19 pandemic. This may have affected recruitment, as our sample size was less than expected. However, positive results in naming were still obtained. Future research will have to determine whether greater and more extensive improvements might be obtained with a larger sample size. In addition, people with aphasia, on average, did not practice for the full dosage prescribed. This, too, leaves a question for future research of whether greater gains might be obtained with more practice.

The study design did not include a maintenance assessment for all participants postintervention (although individuals in the treatment condition had one maintenance assessment at approximately 6 wk postintervention). This is recognized as a limitation, as long-term maintenance of treatment gains is an important outcome in aphasia rehabilitation. Finally, the nature of this study did not allow for participants to be blinded to the condition. However, all individuals completing assessments at each time point were blinded to participant condition. Future studies could examine the efficacy of training using the VoiceAdapt app compared to other speech-language therapy apps on improving naming performance in individuals with aphasia.

### Conclusions

In this RCT, 5 weeks of training in naming single words with the VoiceAdapt app resulted in measurable gains in naming and communication-based quality of life, in contrast to no training in a group of participants in the chronic stages of poststroke aphasia. These changes were seen in a remotely delivered program, in participants who were no longer receiving speech therapy and who practiced their exercises at home using the app.

The findings inform the profession on telerehabilitation practices and monitoring app–based home therapy programs, with a strong recommendation to incorporate UCD methodologies for improved user adherence and therapeutic efficacy [32,55]. In addition, the improvements seen in naming and communicative functioning suggest that instantiating evidence-based treatments such as the one done with this app and which can be found in some others (eg, Tactus and Constant Therapy) can be recommended as an efficacious, easily accessible, and cost-effective method for individuals who are in the chronic stages of recovery and who often have limited options for rehabilitation.

## Supplementary material

10.2196/67711 Checklist 1 CONSORT eHEALTH (V 1.6.1) checklist.

## Abbreviations

ASR: automatic speech recognition
BNT: Boston Naming Test
CETI: Communication Effectiveness Index
PCA: Phonological Components Analysis
RCT: randomized controlled trial
REDCap: Research Electronic Data Capture
SAQoL-39g: Stroke and Aphasia Quality of Life Scale - 39
SFA: Semantic Features Analysis
SLP: speech-language pathologist
SSNT: Study Stimulus Naming Test
SUS: System Usability Scale
UCD: user-centered design
WAB-R: Western Aphasia Battery-Revised
